# Semaglutide attenuates myocardial ischemia-reperfusion injury by inhibiting ferroptosis of cardiomyocytes via activation of PKC-S100A9 axis

**DOI:** 10.3389/fphar.2025.1529652

**Published:** 2025-03-20

**Authors:** Yan Liu, Zixuan Li, Xinhe Xu, Yan Zou, Miaomiao Zhang, Yingyu Chen, Wenwu Zhu, Bing Han

**Affiliations:** ^1^ Xuzhou Clinical College of Xuzhou Medical University, Division of Cardiology, Xuzhou Central Hospital, Xuzhou, Jiangsu, China; ^2^ Xuzhou Institute of Cardiovascular Disease, Xuzhou, Jiangsu, China

**Keywords:** semaglutide, myocardial ischemia-reperfusion injury, S100A9, ferroptosis, cardiovascular

## Abstract

**Objective:**

The incidence of ischemic cardiomyopathy increases annually worldwide, and it is the leading cause of mortality in China. Although interventional diagnostic and therapeutic techniques can promptly open the culprit vessels, myocardial ischemia-reperfusion injury (MIRI), resulting from restored blood flow, is often inevitable. Semaglutide (Sem), a novel GLP-1 analogue, is primarily utilized in managing Type 2 diabetes mellitus (T2DM). Recent research indicates that semaglutide may reduce the risk of major adverse cardiovascular events. Therefore, the purpose of this study is to explore whether semaglutide can ameliorate MIRI and explore its potential mechanism.

**Methods and results:**

: A mouse model of myocardial ischemia-reperfusion (I/R) was created by ligating the left anterior descending coronary artery (LAD) first for 45 min and then reperfusing the heart for 24 h. Assessment of cardiac function and fibrosis were conducted through small animal ultrasound and Masson’s staining. It was observed that semaglutide enhanced cardiac function recovery and diminished fibrosis in the I/R model. *In vivo* experiments, semaglutide proved to mitigate oxidative stress and inhibit ferroptosis in cardiomyocytes. RNA sequencing showed that S100 calcium binding protein A9 (S100A9) was the target gene of semaglutide to protect against MIRI. *In vitro*, experiments showed that semaglutide decreased the expression of S100A9 by activating the Protein Kinase C(PKC) pathway, thus inhibiting ferroptosis in cardiomyocytes.

**Conclusion:**

Semaglutide can reduce I/R-induced myocardial injury by inhibiting the ferroptosis of cardiomyocytes. In the mechanism, semaglutide mainly reduce the expression of S100A9 via the activation of PKC signaling pathway. Therefore, semaglutide is considered as a potential treatment option for MIRI.

## Graphical Abstract

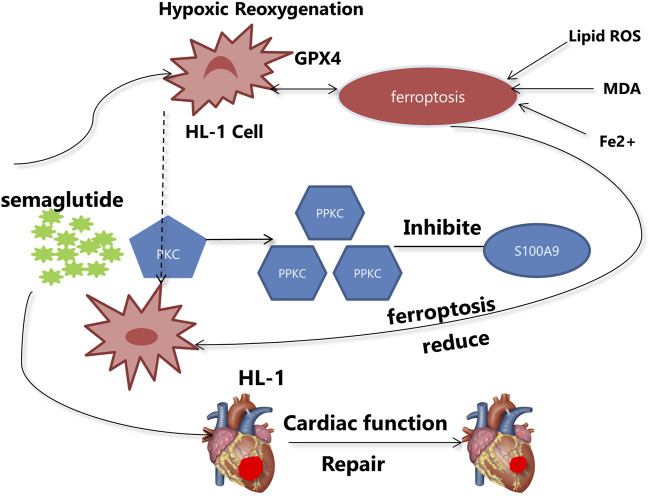

## 1 Introduction

According to the Cardiovascular Health and Diseases Report in China, the number of people in China suffering from ischemic heart disease is as high as 11.39 million, accounting for the highest disease mortality rate (Wang et al., 2023). Although timely percutaneous coronary intervention, thrombolytic therapy, and coronary artery bypass grafting have greatly reduced the complications and risk of death in acute myocardial infarction (AMI), it is inevitable to encounter MIRI during the process of blood flow restoration (Zhang et al., 2024). However, MIRI will still occur during reperfusion (Heusch, 2020). The mechanism of MIRI is complex and involves multiple mechanisms, including inflammatory response (Wu et al., 2021a), increased production of reactive oxygen species (ROS) (Wu et al., 2020), and calcium overload (Wu et al., 2021b), mitochondrial dysfunction (Xu et al., 2019), potentially leading to severe hemodynamic disturbances and endangering patients' lives. Therefore, it is urgent and important to explore new drug therapies and specific molecular mechanisms for the treatment of I/R injury.

There have been reports suggesting that ferroptosis plays a significant role in the occurrence and development of MIRI (Wang et al., 2022; Yang and Lin, 2022; Wang et al., 2022; Yang and Lin, 2022). It was reported in Advanced Science that MIRI promoted the ferroptosis, which severely impaired cardiac function recovery. Ferroptosis, a recently identified form of programmed cell death, has become a focus in cell death research (Dixon et al., 2012). The main mechanism of ferroptosis is the catalysis of highly expressed unsaturated fatty acids on cell membranes by divalent iron or lipoxygenases, leading to lipid peroxidation and ultimately inducing cell death (Yu et al., 2021). Studies also link ferroptosis to various cardiovascular diseases, including doxorubicin-induced cardiac injury, heart failure, and stroke (Tadokoro et al., 2020; Kitakata et al., 2022; Stockwell et al., 2017; Zhang et al., 2023; Urquhart and Willis, 2020). During ferroptosis, there is a reduction in the core enzyme glutathione peroxidase 4 (GPX4), which is involved in the regulation of the antioxidant system. Consequently, myocardial I/R triggers severe oxidative stress, leading to an accumulation of oxygen radicals and ROS, thus accelerating membrane and organelle dysfunction. Therefore, inhibiting myocardial cell ferroptosis may enhance cardiac function recovery (Chen et al., 2023).

Semaglutide, an incretin produced by intestinal L-cells, is primarily utilized for blood glucose regulation in patients with T2DM (Marso et al., 2016). In addition, semaglutide reduces the likelihood of serious adverse cardiovascular events in individuals with this condition. Semaglutide is a human glucagon-like peptide-1 (GLP-1) analogue. Its half-life is about a week, which means that its concentration in the body can be maintained for about a week. Because it has a long half-life and can reduce kidney clearance by binding to albumin in plasma, it can also resist the degradation of endogenous dipeptidease-4 (DDP-4), thus increasing the stability of the drug, so it only needs to be injected once a week to achieve the required therapeutic effect. Currently, there are studies indicating that semaglutide can activate the PI3K/AKT signaling pathway to decrease the expression level of BNIP3, thereby improving mitochondrial function in myocardial cells and alleviating doxorubicin-induced myocardial injury (Li et al., 2024). Additional studies suggest that semaglutide prevents myocardial cell apoptosis by activating the ERK pathway (Zhu et al., 2023). However, it remains uncertain whether semaglutide can alleviate MIRI by suppressing myocardial cell ferroptosis. Thus, the purpose of our study is to investigate the mechanisms by which semaglutide may reduce MIRI.

## 2 Materials and methods

### 2.1 Mouse myocardial I/R model

The male C57BL/6J mice (6–8 weeks), which are healthy and weigh around 20 ± 2 g, were acquired from the Experimental Animal Center of Xuzhou Medical University. Both food and water were freely available to them. For the I/R model, male C57BL/6J mice were numbed with an intraperitoneal injection of sodium pentobarbital (50 mg/kg). A 110 breaths per minute ventilator was used to intubate the mouse through the mouth. Following a thoracotomy at the fourth intercostal gap on the left side, the LAD was ligated under a microscope. Electrocardiogram findings of substantial elevation of the ST segment, diminished pulse strength, and noticeable whiteness of the heart near the ligation site were indicators of a well-established model. The LAD was ligated 45 min and re-perfused 24 h. In the treatment group, semaglutide (0.6 mg/kg; Novo Nordisk Denmark) was administered subcutaneously once weekly for 4 weeks prior to the I/R model establishment. In the sham group, a similar procedure was undertaken, including threading through the LAD, but without the ligation. The following day, post-anesthesia, the hearts were excised via thoracotomy for evaluation using 2, 3, 5-triphenyltetrazolium chloride (TTC) (Sigma, United States) and Evans blue double staining to assess the myocardial infarct (white area) and reperfusion injury (red risk area) in each group. During reperfusion, the mice were monitored frequently to ensure they had adequate food, water, and living conditions.

### 2.2 Cell culture and hypoxia/reoxygenation model

In a Petri dish with 10% fetal bovine serum (FBS), 1% penicillin/streptomycin (PS), 100 μmol/L norepinephrine, and 4 mmol/L L-glutamine (Claycomb medium, 51800C-500mL, Sigma-Aldrich), the HL-1 cells (Catalogue numbers: SCC065, Sigma-Aldrich:St. Louis, MO, United States) were grown with 37°C, 5% CO_2_ and 21% O_2_. After reaching 80% confluency, the cells were randomly divided into the following groups: control group, H/R group, H/R + Sem group (5 mmol/L, added to the culture medium at the beginning of hypoxia). Each group had 3 replicate wells. Hypoxia was induced by incubating the cells in a glucose-free medium under conditions of 5% CO2, 95% N2, 37°C for 4 h, followed by reoxygenation in normal Claycomb medium under conditions of 5% CO_2_, 21% O_2_, 37°C for 4 h.

### 2.3 Tissue collection

Evans blue double staining and TTC were used to assess the region of myocardial infarction. After 24 h of reperfusion, the mice were injected intraperitoneally with anesthetic, and Evans blue was inserted into the aorta. Five sections, each 1–2 mm thick, were cut from the heart after it was quickly frozen at −80°C for around 5 min after extraction. After 30 min in a 2% TTC solution at 37°C, the slices were fixed in a 4% paraformaldehyde solution for 24 h. Using a digital camera, white photographs of the infarct tissue, red images of the perfusion tissue, and blue images of the normal tissue were captured. The myocardial infarct area (%) = [infarct tissue/(infarct tissue + reperfusion tissue)] × 100%. Image analysis was conducted using ImageJ software.

### 2.4 Echocardiography

In order to do the echocardiography, VisualSonics of Toronto, Canada, used their Vevo 2100 ultrasound machine (Chen et al., 2019). To keep the heart rate of the mice between 430 and 480 beats/min, they were momentarily sedated with a mixture of 1.5% isoflurane and 98.5% oxygen. Recordings from at least three consecutive heartbeats were averaged. B- and M-mode short-axis images of the parasternal region were captured. Functional parameters such as left ventricular ejection fraction (LVEF), left ventricular shortening fraction (LVFS), stroke volume (SV), and cardiac output (CO) were calculated using Vevo LAB software (VisualSonics).

### 2.5 Histological analysis

The left ventricle’s transverse sections were cut into 4 μm slices, kept at 4°C for 1 day in 4% paraformaldehyde, dehydrated in ethanol, cleaned in xylene, and finally embedded in paraffin. To observe changes in histology, hematoxylin and eosin staining was used. Tunal staining was employed to assess apoptosis. Masson’s trichrome, Sirius Red staining, and col1, col3 fluorescence staining were utilized to examine cardiac fibrosis. The size of the myocardium was measured using CX43+WGA double staining. To assess ferroptosis levels, fluorescence staining with GPX4 and prostaglandin-endoperoxide synthase 2 (PTGS2) was carried out. Image measurement was carried out using ImageJ software.

### 2.6 Cell surface area measurement

Nuclei were counterstained with 4′, 6-diamidino-2-phenylindole (DAPI), and α-actinin antibodies were used to stain the cardiac tissue of mice (G1012, Servicebio China). We used ImageJ software to quantify cell size after staining with TRITC-phalloidin.

### 2.7 Immunofluorescence staining

Antigen retrieval was performed using Ethylenediaminetetraacetic Acid (EDTA) after deparaffinization and rehydration. Next, the sections were incubated with 3% BSA for half an hour. Proteintech Sanying Biotechnology Co., Ltd. of Wuhan, China, supplied the anti-GPX4 (1:400, ab2909469), PTGS2(COX2) (1:300, ab2881731), and Col1/Col3 (1:2000/1:200, ab3073715) primary antibodies, which were incubated at 4°C overnight. In a dark room, the matching secondary antibodies were left to incubate for half an hour. CY3 flag goat anti-mouse IgG (1:300, ab2923552) and goat anti-rabbit IgG (1:500/1:400, ab2811189/ab2910224) were the antibodies. To counterstain the nuclei, DAPI was used. Olympus Fluoview FV300 software, version 3C, was used to process photomicrographs taken with a Nikon ECLIPSE C1 upright fluorescent microscope. The fluorescence intensity was quantitatively analyzed using the ImageJ program.

### 2.8 Measurement of ferroptosis-related indicator

Lipid peroxidation levels were determined in HL-1 heart cells and cardiac tissues using a Malondialdehyde (MDA) Assay Kit (E-BC-K028-M/E-BC-K025-M). To determine the concentrations of ferrous iron (Fe^2+^) in HL-1 heart cells and animal tissues, we utilized the Cellular Fe^2+^ Colorimetric Assay Kit (E-BC-K881-M) and the Animal Ferrous Iron Colorimetric Assay Kit (E-BC-K773-M) manufactured by Elabscience Biotechnology China. The Superoxide Dismutase (SOD) Assay Kit (S0101S, Beyotime China), Reduced Glutathione (GSH) Colorimetric Assay Kit (E-BC-K030-M Elabscience Biotechnology China) was utilized to evaluate the amounts of SOD, GSH in HL-1 heart cells and animal cardiac tissues. To assess the amounts of ROS in HL-1 cardiac cells, a ROS Detection Kit (S0033M, Beyotime China) was utilized. In six-well plates, the cells were cultivated until they reached 80% confluence. Following treatment, the cells were incubated at 37°C for 20 min in a serum-free medium that included 10 μmol/L DCFH-DA diluted 1:1000. Cells were seen using a confocal laser scanning microscope. All kits were used according to the manufacturer’s recommendations.

### 2.9 Transmission electron microscopy

Myocardial cells from the I/R and I/R + Sem groups were examined for mitochondrial ultrastructure using TEM. The hearts were treated to an electron microscopy fixative at 4°C before being fixed in 1% osmium tetroxide at room temperature for 2 h in total darkness. A solution of 2% uranyl acetate and 2.6% lead citrate was used to make ultrathin sections (60 nm) and stain them. Afterwards, transmission electron microscopy was used to detect and record the mitochondrial morphology.

### 2.10 Western blot analysis

Using RIPA lysis buffer, the total protein was recovered from either cultivated HL-1 cells or tissues of the left ventricle. The proteins were analyzed by Western blotting in accordance with the protocol described by Zou et al. (2022). Proteins were resolved by 10% SDS-polyacrylamide gel electrophoresis after being transferred (30 μg each sample) to PVDF membranes (Millipore, Darmstadt, Germany). The membranes were incubated with primary antibodies at 4°C for the night after being blocked with Western blocking buffer (CWbio, Taizhou, China) for 1 h. A 1-h incubation with HRP was performed on the secondary antibodies at room temperature. For protein detection, an affinity-made enhanced chemiluminescence kit was utilized. The kit was manufactured in Ancaster, Ontario, Canada. Band intensities were quantified using T-anon image analysis software (T-anon, Research article Cell Biology), with β-Actin serving as a loading control.

### 2.11 Real-time fluorescence quantitative PCR

Following the manufacturer’s procedure, total RNA was extracted from tissues of the HL-1cells using an extraction kit (G3013, China). The High Capacity cDNA Reverse Transcription Kit (G337, China) was used for reverse transcription with 200 ng RNA. The experiment was carried out using a real-time quantitative PCR (Rt-qPCR) device developed by Beijing Dongsheng Innovation Biotechnology Co., Ltd. (ETC811). The expression of genes was normalized using GAPDH as a reference gene. The following table provides the primer sequences for qPCR.

### 2.12 RNA-seq analysis

Mice in the I/R and I/R + Sem groups had their cardiac RNA collected for subsequent testing. Genome sequencing, library building, quality control, and RNA isolation were all handled by Guangzhou Jidiao Biotechnology. Illumina Novaseq 6000 was used for library sequencing. We used edgeR software for bioinformatics analysis and DESeq2 for differential expression analysis.

### 2.13 Statistical analysis

We displayed mean ± SD for each data point. A two-tailed test was employed to assess the differences in mean values between the two groups, and single-factor design was used for multi-group anova. We used GraphPad Prism 8 to analyze the data, and we regarded p < 0.05 to be statistically significant.

## 3 Results

### 3.1 Semaglutide inhibits ferroptosis in HL-1 cells in a H/R model

**FIGURE 1:**
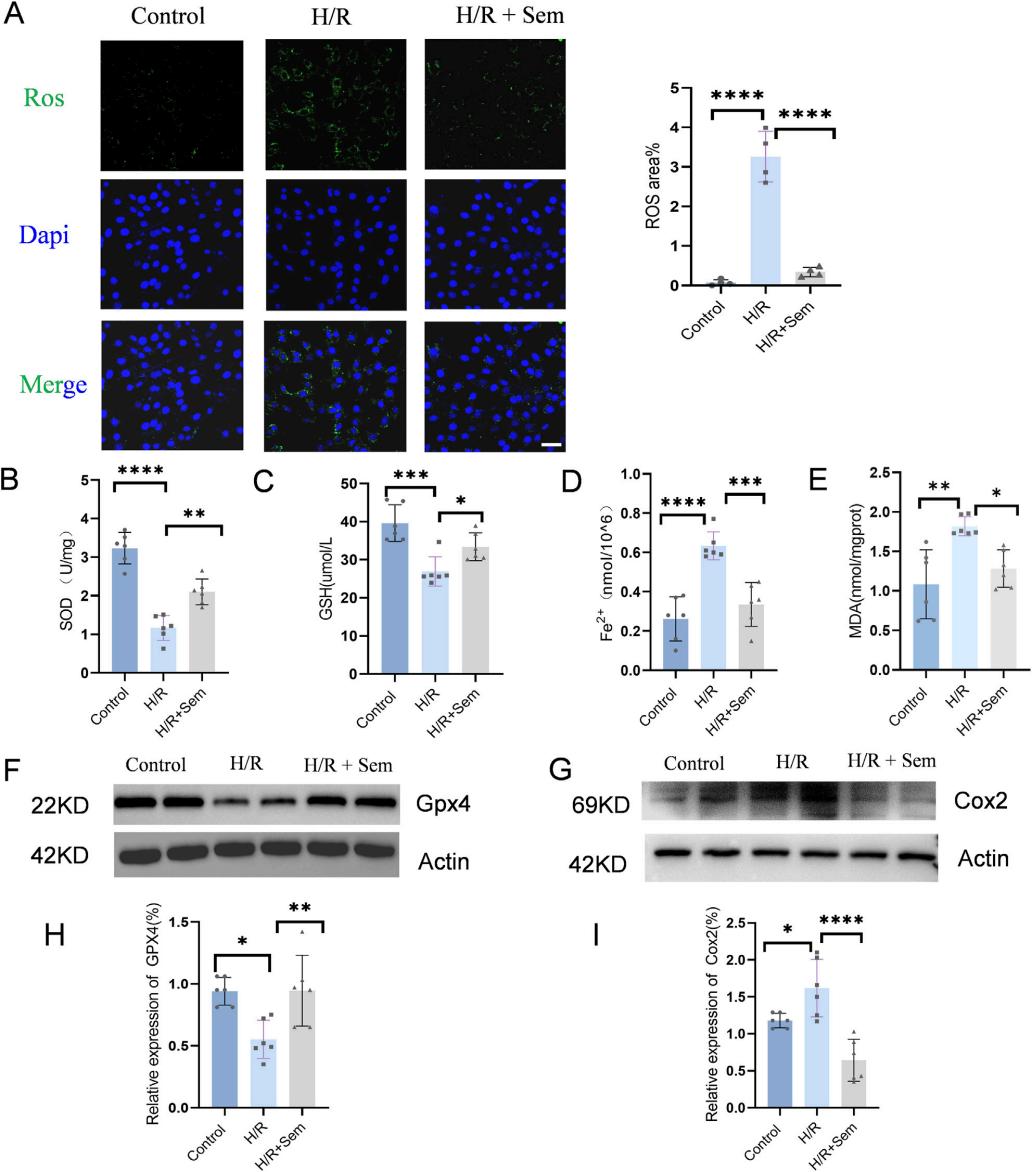
Semaglutide Inhibits Ferroptosis in HL-1 Cells in a H/R Model **(A)** DCFH-DA fluorescent probe was used to detect cellular ROS levels. Scale bar = 25 μm **(B)** WST-8 method was used to detect total SOD activity **(C–E)** Colorimetric method was used to detect reduced glutathione (GSH), cellular ferrous ion (Fe^2+^), and lipid peroxidation marker malondialdehyde (MDA). **(F, H)** Western blot detection of GPX4 protein expression in HL-1 cardiomyocytes of the three groups, using β-Actin as internal control. **(G, I)** Western blot detection of Cox2/PTGS2 protein expression in HL-1 cardiomyocytes of the three groups, using β-Actin as internal control, *p < 0.05; * *p < 0.01; ** *p < 0.001; * ** *p < 0.0001 (n = 6 per group).

A H/R scenario was established using the HL-1 cardiomyocyte line in order to evaluate the protective effect of semaglutide. It was observed that semaglutide significantly reduced ROS levels in cardiomyocytes compared to the H/R group (Figure 1A). Additionally, semaglutide markedly elevated the expression levels of intracellular antioxidants GSH and SOD, compared to the ischemia-hypoxia group (Figures 1B, C). Further findings revealed that semaglutide decreased the levels of Fe^2+^ and MDA in HL-1 cells, indicating its potential to protect HL-1 cells by inhibiting cardiomyocyte ferroptosis (Figures 1D, E). Western blot analysis verified a reduction in GPX4 expression due to ischemia-hypoxia, while semaglutide significantly enhanced GPX4 levels (Figures 1F, H). Additionally, the results showed that compared to the ischemia-hypoxia group, semaglutide significantly reduced COX2 expression (Figures 1G, I). These findings provide preliminary evidence that semaglutide protects cardiomyocytes from damage caused by H/R.

### 3.2 Semaglutide reduces I/R myocardial injury and promotes cardiac function recovery

**FIGURE 2:**
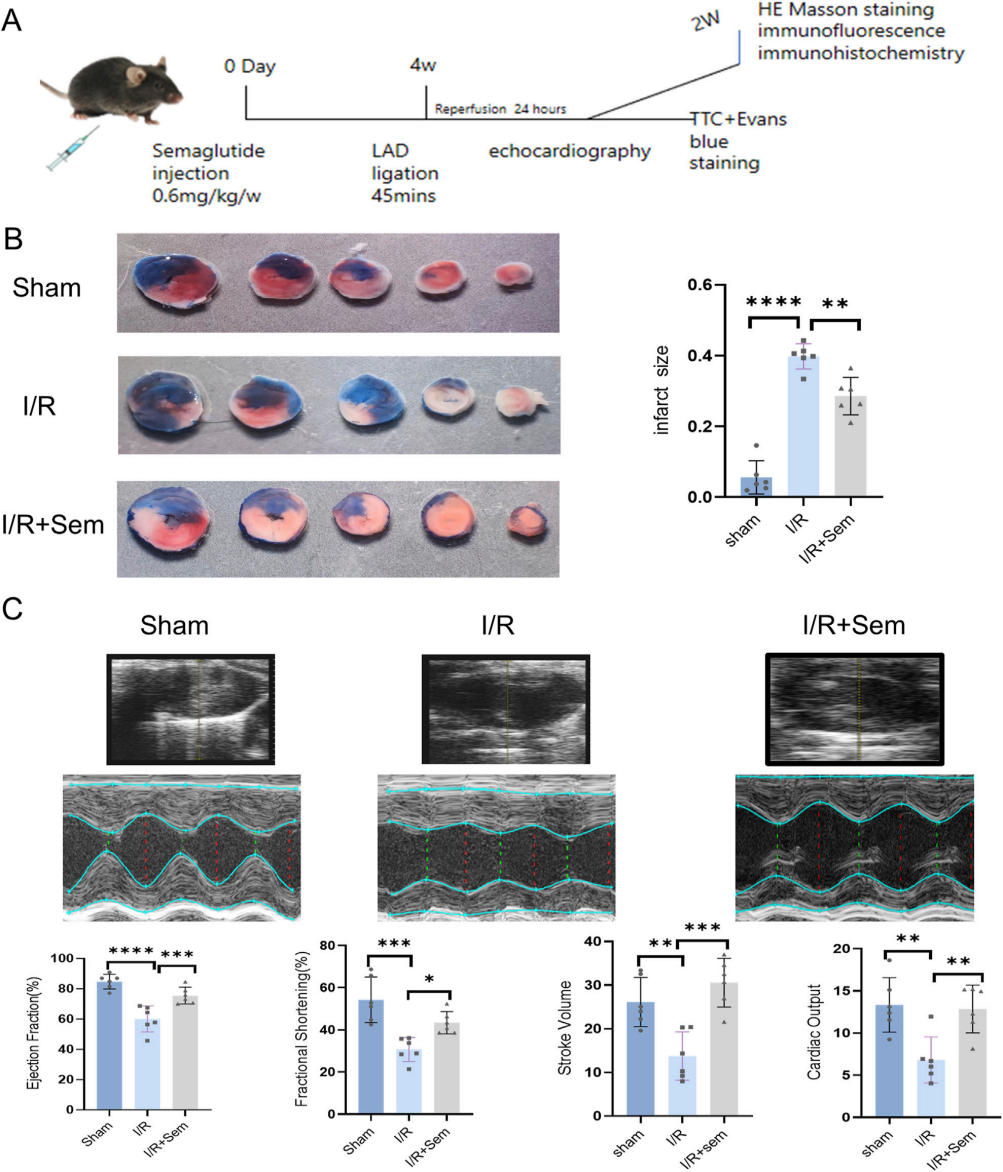
Semaglutide Reduces I/R Myocardial Injury and Promotes Cardiac Function Recovery **(A)** experimental model of myocardial ischemia-reperfusion injury in animals **(B)** representative images of infarct size detected by TTC staining and quantitative data of cardiac infarct size in mice **(C)** echocardiography of mice 1 day after surgery, cardiac function indicators:LVEF (left ventricular ejection fraction), LVFS (left ventricular shortening fraction), SV (stroke volume), and CO(cardiac output),*p < 0.05; * *p < 0.01; ** *p < 0.001; * ** *p < 0.0001 (n = 6 per group).

The cellular results informed the adoption of a MIRI mouse model to investigate semaglutide’s impact on cardiac function. As illustrated in Figure 2A, a cardiac I/R model was developed using mice. Infarct areas, LVEF, LVFS, SV, and CO were significantly different between the sham and I/R groups. In the TTC trials, semaglutide group showed a marked reduction in infarct size and a considerable improvement in LVFS, LVEF, CO and SV, whereas the I/R group showed a marked increase in infarct size (Figures 2B, C). These preliminary results suggest that semaglutide can improve I/R injury and promote cardiac function recovery.

### 3.3 Semaglutide inhibits ferroptosis in an *in vivo* myocardial I/R model

**FIGURE 3:**
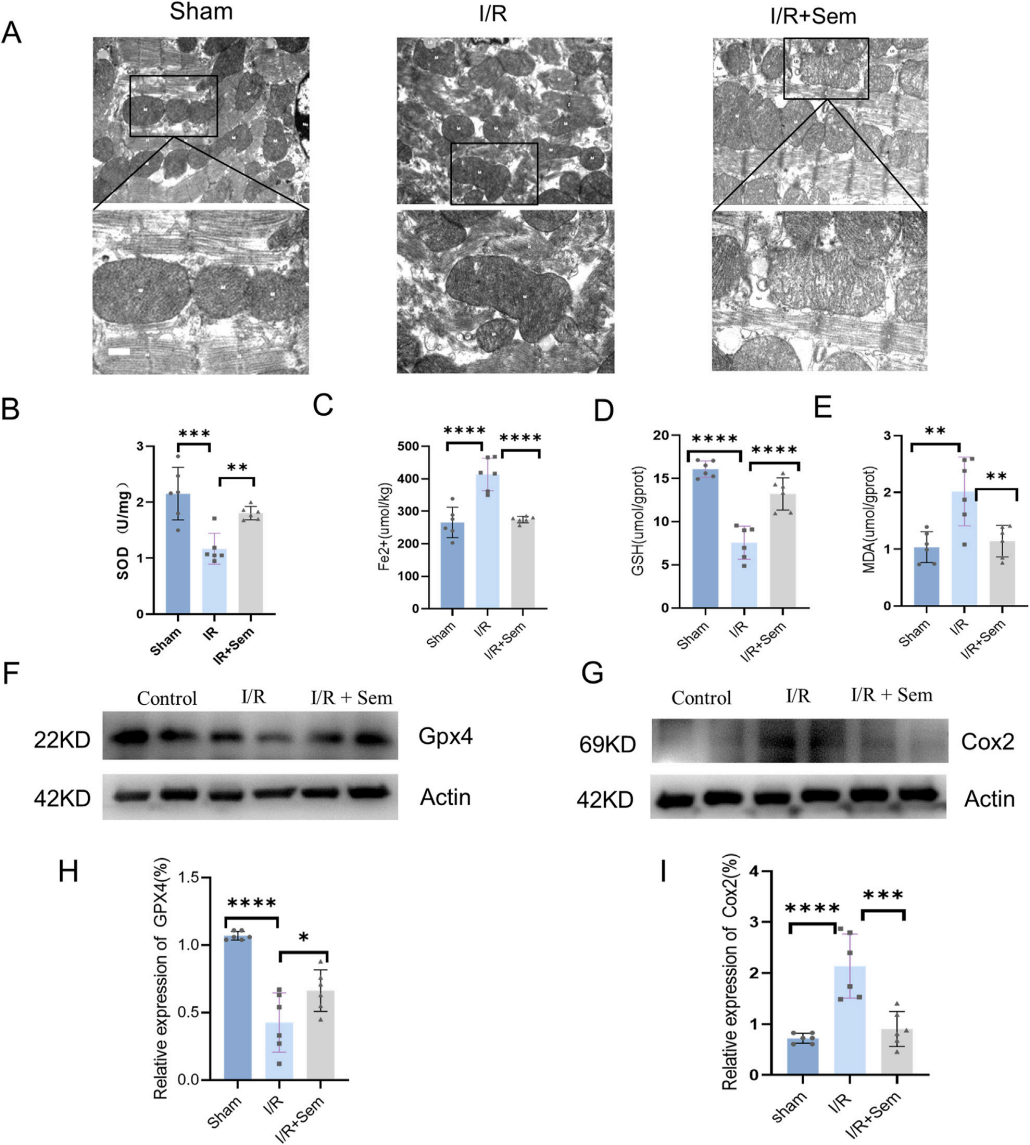
Semaglutide Inhibits Ferroptosis in an *In Vivo* Myocardial I/R Model **(A)** Mitochondrial morphology associated with iron death was observed under transmission electron microscopy. Scale bar = 25 μm **(B)** Total SOD activity in mouse myocardial tissue was detected by WST-8 method. **(C–E)** Reduced ferrous ion (Fe^2+^), glutathione (GSH), and malondialdehyde (MDA), a lipid peroxidation marker, were detected by colorimetry. **(F, H)** Western blot detection of GPX4 protein expression in myocardial tissue of mice in the three groups, and β-Actin was used as internal control for sample analysis. **(G, I)** Western blot detection of Cox2/PTGS2 protein expression in myocardial tissue of mice in the three groups, and β-Actin was used as internal control for sample analysis, *p < 0.05; * *p < 0.01; ** *p < 0.001; * ** *p < 0.0001 (n = 6 per group).

**FIGURE 4:**
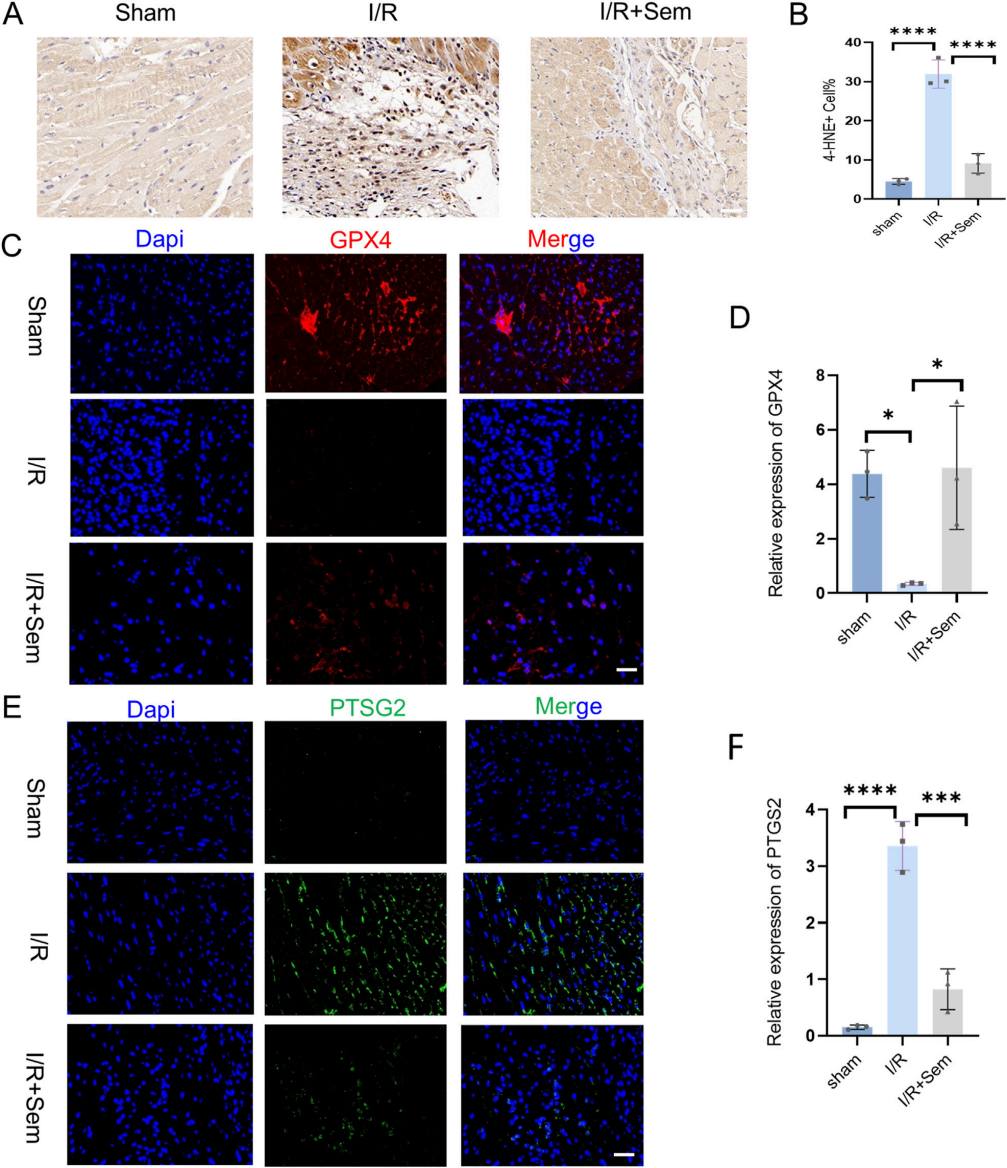
Semaglutide Inhibits Ferroptosis in an *In Vivo* Myocardial I/R Model **(A, B)** Sham operation group, ischemia-reperfusion group, ischemia-reperfusion + Sem group 4-HNE Immunohistochemistry and statistical diagram of ischemia-reperfusion mouse heart tissue. Scale bar = 25 μm. **(C, D)** Sham operation group, ischemia-reperfusion group, ischemia-reperfusion + Sem group, GPX4 staining image and statistical diagram, Red representsGPX4; Blue represents DAPI-stained nuclei. Scale bar = 25 μm **(E, F)** PTGS2/Cox2 staining image and statistical diagram of heart tissue of ischemia-reperfusion mice in sham operation group, ischemia-reperfusion group, ischemia-reperfusion + Sem group, Green represents PTGS2; Blue represents DAPI-stained nuclei, Scale bar = 25 μm,*p < 0.05; * *p < 0.01; ** *p < 0.001; * ** *p < 0.0001 (n = 3 per group).

The fact that semaglutide reduces I/R damage has been proven in both animal and cell studies. The development of cardiac I/R damage is dependent on mitochondria. The ultrastructure of cardiomyocyte mitochondria was examined using transmission electron microscopy. As shown in Figure 3, after MIRI, there is mitochondrial membrane shrinkage and a decrease in the clarity of mitochondrial cristae. Mitochondrial membrane integrity and cristae visibility were both improved in the semaglutide group as compared to the I/R group (Figure 3A). Also, in comparison to the sham group, the I/R group’s myocardial SOD and GSH activity was much lower, and their MDA release and Fe^2+^ concentration were much higher. Myocardial SOD and GSH activity was much enhanced in the semaglutide group, although MDA release and Fe^2+^ concentration were significantly decreased (Figures 3B–E). The Western blot analysis revealed that the semaglutide group had much higher GPX4 protein levels and much lower COX2 protein expression when compared with the I/R group (Figures 3F–I). The immunohistochemical results demonstrated a significant reduction in 4-HNE in the semaglutide group compared to the I/R group (Figures 4A, B). In addition, compared to the I/R group, the semaglutide group displayed higher levels of GPX4 fluorescence expression and lower levels of PTGS2(COX2) fluorescence staining in cardiac tissue (Figures 4C–F). These findings suggest that semaglutide may protect against I/R damage by avoiding ferroptosis.

### 3.4 Semaglutide reduces myocardial fibrosis and apoptosis in an *in vivo* I/R model

**FIGURE 5:**
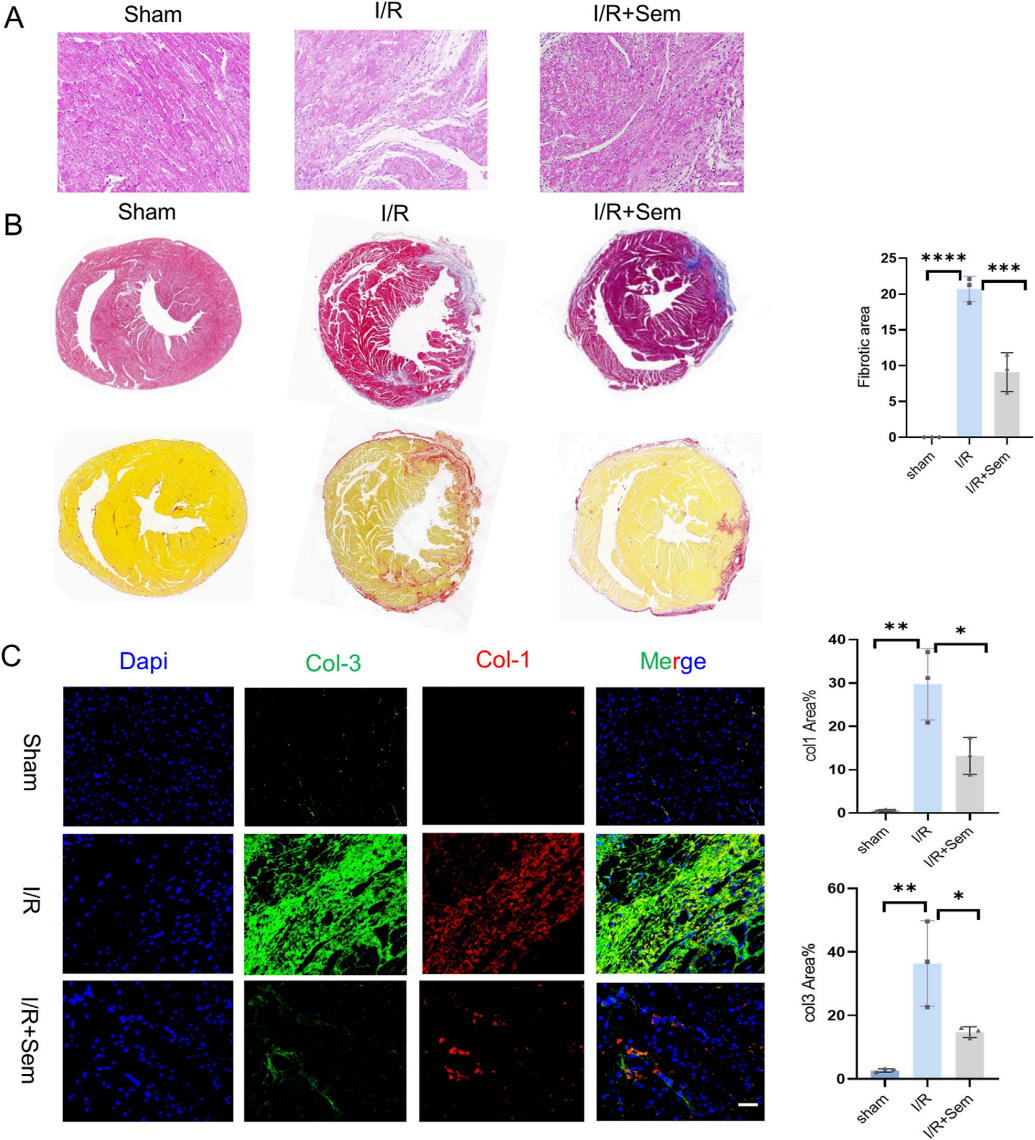
Semaglutide Reduces Myocardial Fibrosis and Apoptosis in an *In Vivo* I/R Model **(A, B)** 14 days after myocardial ischemia-reperfusion injury with eosin (HE) and hematoxylin staining and quantitative analysis of cardiac fibrosis in each experimental group. Scale bar = 25 μm **(C)** sham operation group, Col1, Col3 staining images and statistical charts of heart tissue of mice with ischemia-reperfusion injury and Sem + ischemia-reperfusion injury group, Red represents Col1, Green is Col3, Blue represents DAPI-stained nuclei. Scale bar = 25 μm *p < 0.05; * *p < 0.01; ** *p < 0.001; * ** *p < 0.0001 (n = 3 per group).

**FIGURE 6:**
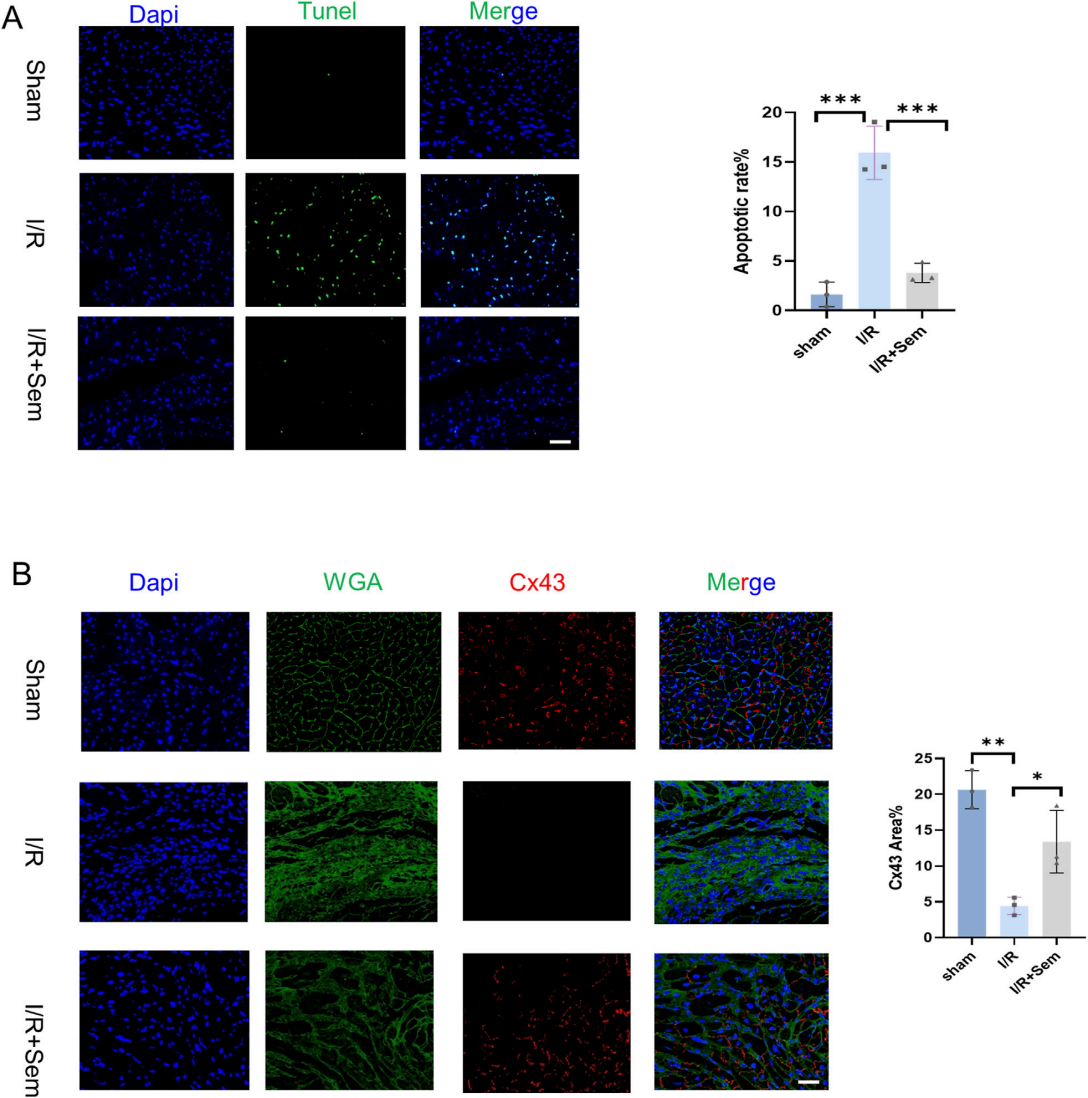
Semaglutide Reduces Myocardial Fibrosis and Apoptosis in an *In Vivo* I/R Model **(A)** TUNEL staining images and statistical graphs of cardiac tissue of myocardial ischemia-reperfusion mice treated by sham operation group, ischemia-reperfusion group, and Sem + ischemia-reperfusion group. Scale bar = 25 μm **(B)** 14-day boundary sections of each experimental group. The expression of Connexin 43 (Cx43) was detected by immunofluorescence staining. Red represents Connexin 43; Green is WGA; Blue represents DAPI-stained nuclei. Scale bar = 25 μm. Continuous variables were expressed as mean ± SEM, *p < 0.05; * *p < 0.01; ** *p < 0.001; * ** *p < 0.0001 (n = 3 per group).

Histopathological examinations corroborated the functional data. HE stains revealed that the myocardial microstructure was dense and orderly in the sham group, featuring intact myocardial fibers without crack. The cells of the cardiac tissue were highly disordered in the I/R group, and the myocardial fibers were notably enlarged and broken. Myocardial tissue lesions were considerably less in the semaglutide group than in the I/R group (Figure 5A). As shown in Figures 5B, C, compared to the sham group, Masson’s trichrome and Sirius Red staining indicated a significant increase in fibrosis in the I/R group. The fibrosis was notably increased after 2 weeks of I/R. Results showed that semaglutide significantly reduced fibrosis in the semaglutide group, indicating that semaglutide can reduce I/R-induced myocardial fibrosis and improve cardiac protection. Figure 6A shows that the semaglutide group had a substantially decreased apoptosis rate. Figure 6B shows that Cx43 expression levels were comparatively higher in the semaglutide group compared to the I/R group. These results preliminarily confirm that semaglutide effectively reduces myocardial fibrosis and cardiomyocyte apoptosis post-myocardial infarction and elevates Cx43 expression in the heart.

### 3.5 Semaglutide significantly inhibits the expression of S100A9

**FIGURE 7:**
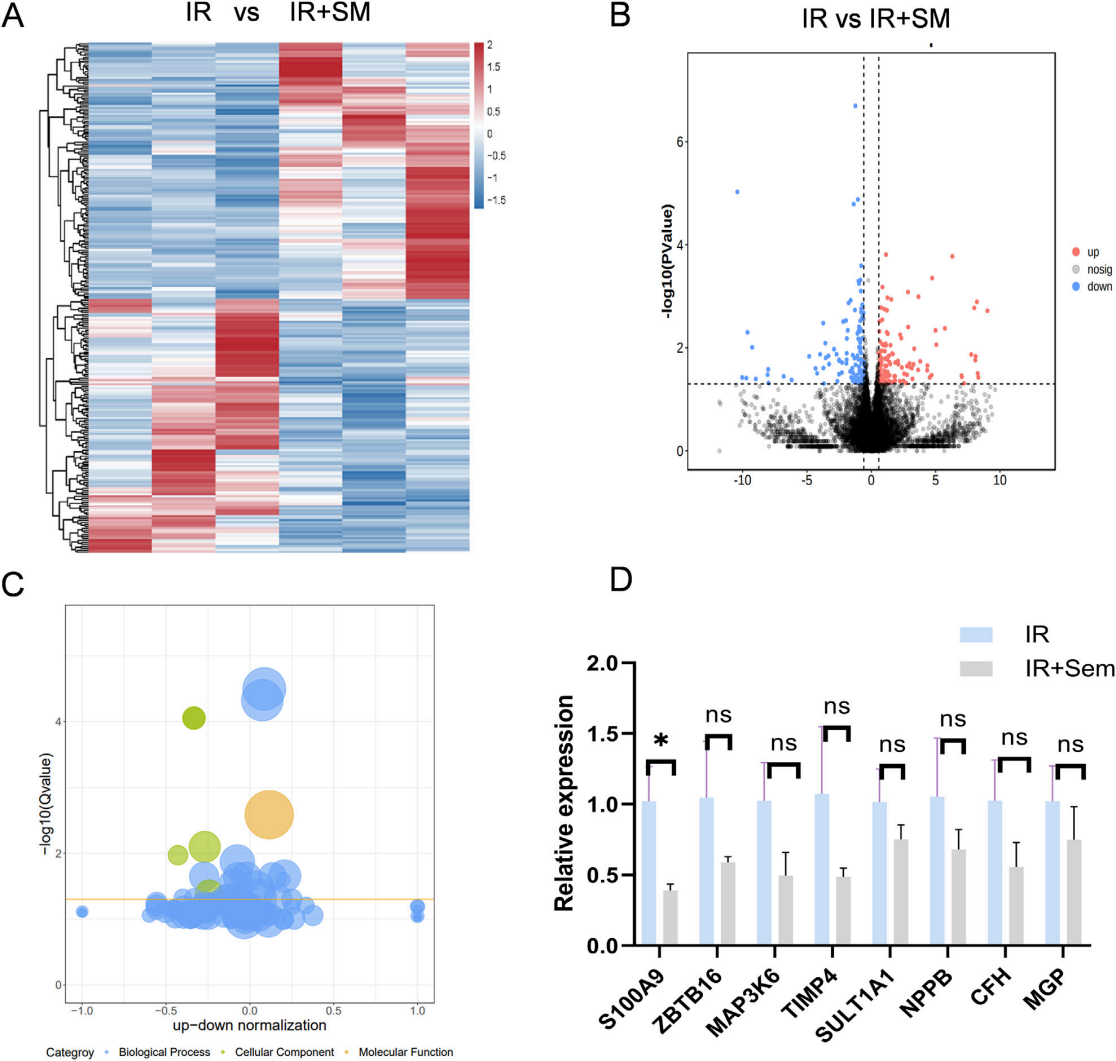
Semaglutide Significantly Inhibits the Expression of S100A9 **(A)** Go-related gene set enrichment analysis (GSEA) to search for functional expression association **(B)** volcano gene expression map between ischemia-reperfusion group and Sem + ischemia-reperfusion group **(C)** Bubble gene expression map between ischemia-reperfusion group and Sem + ischemia-reperfusion group **(D)** ischemia-reperfusion + Sem group qRT-PCR results of 8 differentially expressed genes in ischemia-reperfusion group were compared (n = 3, *p < 0.05).

To delve deeper into the mechanism of semaglutide’s protective effects against I/R injury, RNA-seq sequencing was utilized to examine potential transcriptional changes. 128 genes were found to be upregulated and 127 genes to be downregulated in the semaglutide group as compared to the I/R group. GO-related gene set enrichment analysis (GSEA) was employed to identify functionally related expressions, revealing that the top 10 GO analysis terms were predominantly associated with positive biological processes (Figures 7A–C). Subsequently, the expression of the top eight differentially expressed genes was validated in mouse myocardial tissue using qRT-PCR, showing that S100A9, with significantly reduced expression, exhibited a statistical difference (p < 0.05) (Figure 7D). Previous studies have shown a certain association between S100A9 and the occurrence and development of cardiovascular diseases, which has become a focus of our attention as well (Averill et al., 2012; Marinković et al., 2020; Zhao et al., 2023; Chen et al., 2024).

### 3.6 Semaglutide mitigates MIRI through the PKC-S100A9 pathway

**FIGURE 8:**
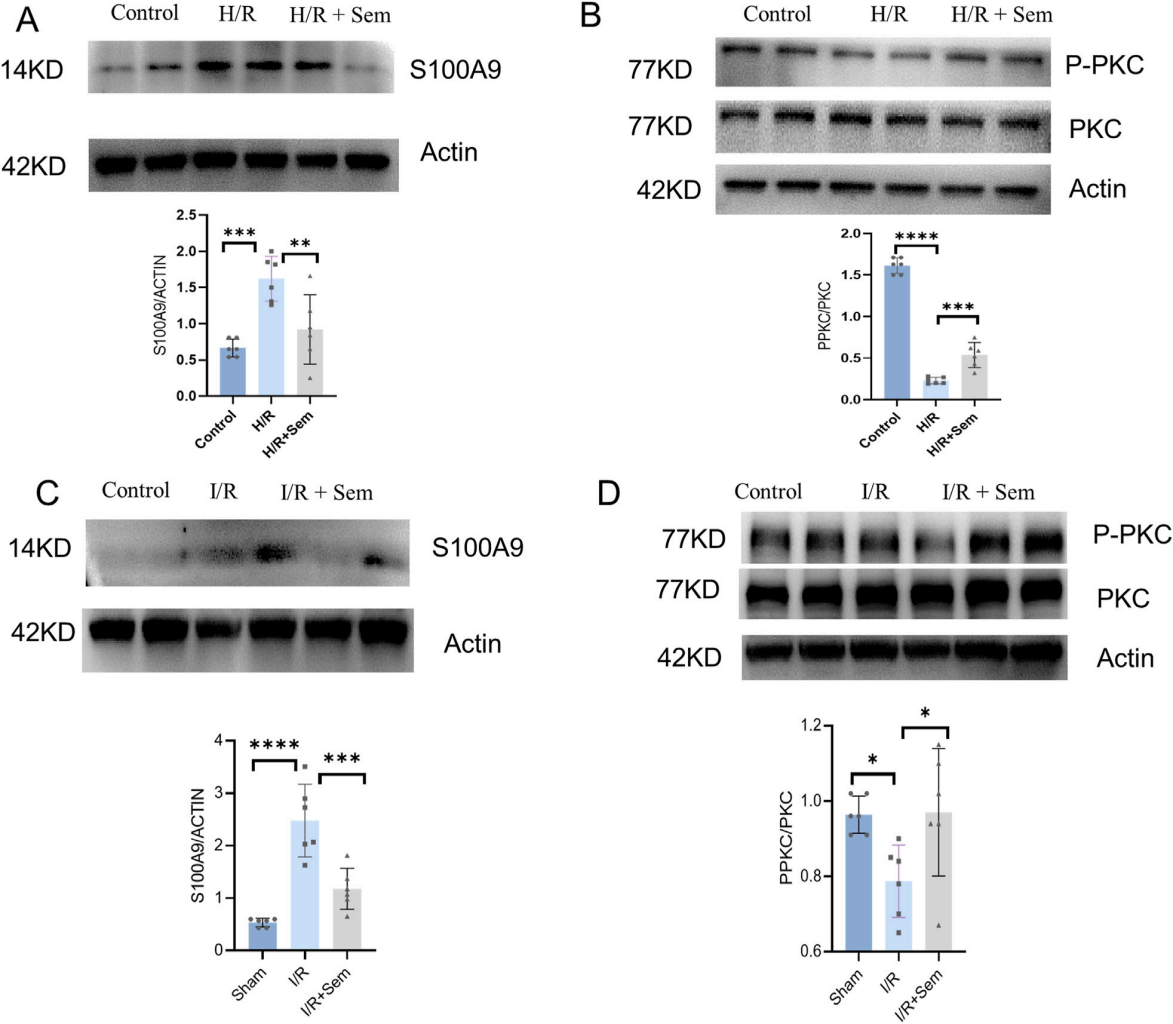
Semaglutide Mitigates MIRI through the PKC-S100A9 Pathway **(A)** Western blot analysis of S100A9 protein in HL-1 cardiomyocytes was detected by using β-Actin as internal control **(B)** Western blot analysis of p-PKC and PKC protein expression in HL-1 cardiomyocytes of the three groups by using β-Actin as internal control **(C)** Western blot analysis of S100A9 protein expression in mouse myocardial tissue by using β-Actin as internal control. **(D)** The expression of p-PKC and PKC protein in the myocardium of the three groups was detected by using β-Actin as internal control *p < 0.05; * *p < 0.01; ** *p < 0.001; * ** *p < 0.0001 (n = 6 per group).

In order to further investigate the signaling pathways involved in the biological activity of semaglutide, we will co-culture semaglutide with cardiomyocytes under H/R conditions. Semaglutide was discovered to raise PKC phosphorylation levels while lowering S100A9 expression levels *in vitro* (Figures 8A, B). Furthermore, *in vivo* experiments, we have also found that semaglutide can increase the expression level of PKC phosphorylation and inhibit the expression level of S100A9 compared to the I/R group (Figures 8C, D). We also found that incubation of semaglutide and S100A9 could significantly lessen the effects of semaglutide on anti-ferroptosis in HL-1 Cells compared with semaglutide and vector (Supplementary Figure 1). Overall, these findings substantiate that semaglutide alleviates MIRI via the PKC-S100A9 pathway.

## 4 Discussion

Ischemic heart disease is prevalent worldwide, and reperfusion injury, resulting from opening the culprit vessel, is inevitable (He et al., 2022). Currently, no effective treatments exist to mitigate I/R injury induced by interventional therapy. In this study, two significant findings were made. First, it was discovered that semaglutide exerts a cardioprotective effect by inhibiting cardiomyocyte ferroptosis, thereby reducing myocardial injury caused by I/R. Second, it was demonstrated that semaglutide inhibits cardiomyocyte ferroptosis via the PKC/S100A9 pathway, unveiling a novel mechanism through which semaglutide lowers the risk of adverse cardiovascular events.

Semaglutide, a long-acting GLP-1A agonist, has received FDA approval for the treatment of T2DM characterized by inadequate glycemic control (Lau et al., 2015). Clinical trials have confirmed its positive cardioprotective effects. Patients suffering from obesity and heart failure who have preserved ejection fraction fare better when given semaglutide, according to the STEP-HFpEF trial (Borlaug et al., 2023; Kosiborod et al., 2023; Borlaug et al., 2023; Kosiborod et al., 2023). Yu-Lan Ma et al. reported that semaglutide enhances ventricular remodeling in mice by optimizing the Creb5/NR4a1 axis (Ma et al., 2024). Xiao-Ping Li et al. observed that semaglutide mitigates doxorubicin-induced cardiotoxicity and supports cardiac function recovery by improving BNIP3-mediated mitochondrial dysfunction (Li et al., 2024). The current study suggests that semaglutide may be a viable option for reducing MIRI, primarily by inhibiting cardiomyocyte ferroptosis. Semaglutide shows promise as a potential treatment for MIRI due to its cardioprotective effects and metabolic benefits. However, further studies are needed to optimize its dosing and timing. It has been reported that investigational drugs such as baicalin (Fan et al., 2021) and cyanidin-3-glucoside (C3G) (Shan et al., 2021) can alleviate MIRI by inhibiting ferroptosis, reducing oxidative stress and Fe^2+^ content, or inhibiting lipid peroxidation. These findings highlight the potential of targeting ferroptosis in the treatment of MIRI. However, a common limitation is that these investigational drugs have not yet been used in clinical practice. Given this gap, our study focused on identifying drugs that are already in clinical use and have the potential to reduce ferroptosis to alleviate MIRI.

Although the mechanisms underlying I/R injury remain incompletely understood, oxidative stress, apoptosis (Zhai et al., 2017), calcium overload (Wu et al., 2021b), and the release of inflammatory mediators (Wu et al., 2021a) are recognized as possible contributors. The current research finds that MIRI exacerbates oxidative stress and induces cardiomyocyte ferroptosis, which semaglutide can alleviate. RNA-Seq sequencing indicated that S100A9 might be a regulated target gene of semaglutide, controlling cardiomyocyte ferroptosis. S100A9, an important component of inflammatory and immunological response pathways, is a calgranulin family member that normally occurs as a heterodimer (Wang et al., 2018; Sreejit et al., 2020; Pan et al., 2024; Jakobsson et al., 2023). Research (Li et al., 2019) has indicated that S100A9 can inhibit mitochondrial respiratory response, exacerbating cellular damage. The expression level of S100A9 was significantly diminished following semaglutide administration, suggesting that S100A9 could be a potential target for semaglutide in alleviating MIRI.

The obesity marker FABP4 induces vascular inflammatory atherosclerosis (García-Vega et al., 2024) and prior research has demonstrated that GLP-1 can affect the course of this disease (Park et al., 2024; Rakipovski et al., 2018; Saraiva and Franco, 2021). Further research has shown that GLP-1 inhibits cardiomyocyte death via activating the PKG/ERK pathway, hence decreasing the fibrosis area in the infarct site (Zhu et al., 2023). The role of the PKC/S100A9 pathway in semaglutide’s mitigation of I/R injury was validated by this research. Additionally, semaglutide was found to activate the PKC pathway according to immunoblotting data. Semaglutide prevents ferroptosis and lowers oxidative stress damage in cardiomyocytes, as confirmed by both *in vivo* and *in vitro* tests. In conclusion, this study showed that semaglutide can reduce cardiac damage after I/R via regulating the PKC/S100A9 pathway and inhibiting ferroptosis in cardiomyocytes.

## 5 Conclusion

In summary, semaglutide can inhibit ferroptosis in cardiomyocytes through the PKC/S100A9 pathway, thereby diminishing myocardial damage following I/R and enhancing cardiac function recovery. Therefore, semaglutide represents a potential novel therapeutic strategy for treating MIRI.

## Data Availability

The data presented in the study are deposited in the Genome Sequence Archive (Genomics, Proteomics & Bioinformatics 2021 [Chen et al., 2021]) in National Genomics Data Center (Nucleic Acids Res 2024 [CNCB-NGDC Members and Partners, 2024]), China National Center for Bioinformation / Beijing Institute of Genomics, Chinese Academy of Sciences repository, accession number “GSA: CRA023327E” that are publicly accessible at https://ngdc.cncb.ac.cn/gsa.

## References

[B1] Averill M. M. Kerkhoff C. Bornfeldt K. E. (2012). S100A8 and S100A9 in cardiovascular biology and disease. Arteriosclerosis, thrombosis, Vasc. Biol. 32 (2), 223–229. 10.1161/ATVBAHA.111.236927 PMC3262097 22095980

[B2] Borlaug B. A. Kitzman D. W. Davies M. J. Rasmussen S. Barros E. Butler J. (2023). Semaglutide in HFpEF across obesity class and by body weight reduction: a prespecified analysis of the STEP-HFpEF trial. Nat. Med. 29 (9), 2358–2365. 10.1038/s41591-023-02526-x 37635157 PMC10504076

[B3] Chen F. He Z. Wang C. Si J. Chen Z. Guo Y. (2024). Advances in the study of S100A9 in cardiovascular diseases. Cell Prolif. 57, e13636. 10.1111/cpr.13636 38504474 PMC11294427

[B4] Chen P. Liu J. Ruan H. Zhang M. Wu P. Yimei D. (2019). Protective effects of Salidroside on cardiac function in mice with myocardial infarction. Sci. Rep. 9 (1), 18127. 10.1038/s41598-019-54713-x 31792327 PMC6888872

[B5] Chen T. Chen X. Zhang S. Zhu J. Tang B. Wang A. (2021). The Genome sequence archive family: toward explosive data growth and diverse data types. Genomics, proteomics & Bioinforma. 19 (4), 578–583. 10.1016/j.gpb.2021.08.001 PMC9039563 34400360

[B6] Chen W. Zhang Y. Wang Z. Tan M. Lin J. Qian X. (2023). Dapagliflozin alleviates myocardial ischemia/reperfusion injury by reducing ferroptosis via MAPK signaling inhibition. Front. Pharmacol. 14, 1078205. 10.3389/fphar.2023.1078205 36891270 PMC9986553

[B7] CNCB-NGDC Members and Partners (2024). Database Resources of the national Genomics data center, China national center for bioinformation in 2024. Nucleic acids Res. 52 (D1), D18–D32. 10.1093/nar/gkad1078 38018256 PMC10767964

[B8] Dixon S. J. Lemberg K. M. Lamprecht M. R. Skouta R. Zaitsev E. M. Gleason C. E. (2012). Ferroptosis: an iron-dependent form of nonapoptotic cell death. Cell 149 (5), 1060–1072. 10.1016/j.cell.2012.03.042 22632970 PMC3367386

[B9] Fan Z. Cai L. Wang S. Wang J. Chen B. (2021). Baicalin prevents myocardial ischemia/reperfusion injury through inhibiting ACSL4 mediated ferroptosis. Front. Pharmacol. 12, 628988. 10.3389/fphar.2021.628988 33935719 PMC8079950

[B10] García-Vega D. Sánchez-López D. Rodríguez-Carnero G. Villar-Taibo R. Viñuela J. E. Lestegás-Soto A. (2024). Semaglutide modulates prothrombotic and atherosclerotic mechanisms, associated with epicardial fat, neutrophils and endothelial cells network. Cardiovasc. Diabetol. 23 (1), 1. 10.1186/s12933-023-02096-9 38172989 PMC10765851

[B11] He J. Liu D. Zhao L. Zhou D. Rong J. Zhang L. (2022). Myocardial ischemia/reperfusion injury: mechanisms of injury and implications for management (Review). Exp. Ther. Med. 23 (6), 430. 10.3892/etm.2022.11357 35607376 PMC9121204

[B12] Heusch G. (2020). Myocardial ischaemia-reperfusion injury and cardioprotection in perspective. Nat. Rev. Cardiol. 17 (12), 773–789. 10.1038/s41569-020-0403-y 32620851

[B13] Jakobsson G. Papareddy P. Andersson H. Mulholland M. Bhongir R. Ljungcrantz I. (2023). Therapeutic S100A8/A9 blockade inhibits myocardial and systemic inflammation and mitigates sepsis-induced myocardial dysfunction. Crit. care London, Engl. 27 (1), 374. 10.1186/s13054-023-04652-x PMC10540409 37773186

[B14] Kitakata H. Endo J. Ikura H. Moriyama H. Shirakawa K. Katsumata Y. (2022). Therapeutic targets for DOX-induced cardiomyopathy: role of apoptosis vs. Ferroptosis. Int. J. Mol. Sci. 23 (3), 1414. 10.3390/ijms23031414 35163335 PMC8835899

[B15] Kosiborod M. N. Abildstrøm S. Z. Borlaug B. A. Butler J. Rasmussen S. Davies M. (2023). Semaglutide in patients with heart failure with preserved ejection fraction and obesity. N. Engl. J. Med. 389 (12), 1069–1084. 10.1056/NEJMoa2306963 37622681

[B16] Lau J. Bloch P. Schäffer L. Pettersson I. Spetzler J. Kofoed J. (2015). Discovery of the once-weekly glucagon-like peptide-1 (GLP-1) analogue semaglutide. J. Med. Chem. 58 (18), 7370–7380. 10.1021/acs.jmedchem.5b00726 26308095

[B17] Li X. Luo W. Tang Y. Wu J. Zhang J. Chen S. (2024). Semaglutide attenuates doxorubicin-induced cardiotoxicity by ameliorating BNIP3-Mediated mitochondrial dysfunction. Redox Biol. 72, 103129. 10.1016/j.redox.2024.103129 38574433 PMC11000183

[B18] Li Y. Chen B. Yang X. Zhang C. Jiao Y. Li P. (2019). S100a8/a9 signaling causes mitochondrial dysfunction and cardiomyocyte death in response to ischemic/reperfusion injury. Circulation 140 (9), 751–764. 10.1161/CIRCULATIONAHA.118.039262 31220942

[B19] Ma Y. L. Kong C. Y. Guo Z. Wang M. Y. Wang P. Liu F. Y. (2024). Semaglutide ameliorates cardiac remodeling in male mice by optimizing energy substrate utilization through the Creb5/NR4a1 axis. Nat. Commun. 15 (1), 4757. 10.1038/s41467-024-48970-2 38834564 PMC11150406

[B20] Marinković G. Koenis D. S. de Camp L. Jablonowski R. Graber N. de Waard V. (2020). S100A9 links inflammation and repair in myocardial infarction. Circulation Res. 127 (5), 664–676. 10.1161/CIRCRESAHA.120.315865 32434457

[B21] Marso S. P. Bain S. C. Consoli A. Eliaschewitz F. G. Jódar E. Leiter L. A. (2016). Semaglutide and cardiovascular outcomes in patients with type 2 diabetes. N. Engl. J. Med. 375 (19), 1834–1844. 10.1056/NEJMoa1607141 27633186

[B22] Pan X. Yang L. Wang S. Liu Y. Yue L. Chen S. (2024). Semaglutide ameliorates obesity-induced cardiac inflammation and oxidative stress mediated via reduction of neutrophil Cxcl2, S100a8, and S100a9 expression. Mol. Cell. Biochem. 479 (5), 1133–1147. 10.1007/s11010-023-04784-2 37318712

[B23] Park B. Bakbak E. Teoh H. Krishnaraj A. Dennis F. Quan A. (2024). GLP-1 receptor agonists and atherosclerosis protection: the vascular endothelium takes center stage. Am. J. physiology. Heart circulatory physiology 326 (5), H1159–H1176. 10.1152/ajpheart.00574.2023 38426865

[B24] Rakipovski G. Rolin B. Nøhr J. Klewe I. Frederiksen K. S. Augustin R. (2018). The GLP-1 analogs liraglutide and semaglutide reduce atherosclerosis in ApoE^-/-^ and LDLr^-/-^ mice by a mechanism that includes inflammatory pathways. JACC. Basic Transl. Sci. 3 (6), 844–857. 10.1016/j.jacbts.2018.09.004 30623143 PMC6314963

[B25] Saraiva J. F. K. Franco D. (2021). Oral GLP-1 analogue: perspectives and impact on atherosclerosis in type 2 diabetic patients. Cardiovasc. Diabetol. 20 (1), 235. 10.1186/s12933-021-01417-0 34911560 PMC8675489

[B26] Shan X. Lv Z. Y. Yin M. J. Chen J. Wang J. Wu Q. N. (2021). The protective effect of cyanidin-3-glucoside on myocardial ischemia-reperfusion injury through ferroptosis. Oxidative Med. Cell. Longev. 2021, 8880141. 10.1155/2021/8880141 PMC7884153 33628391

[B27] Sreejit G. Abdel Latif A. Murphy A. J. Nagareddy P. R. (2020). Emerging roles of neutrophil-borne S100A8/A9 in cardiovascular inflammation. Pharmacol. Res. 161, 105212. 10.1016/j.phrs.2020.105212 32991974 PMC7755830

[B28] Stockwell B. R. Friedmann Angeli J. P. Bayir H. Bush A. I. Conrad M. Dixon S. J. (2017). Ferroptosis: a regulated cell death nexus linking metabolism, redox biology, and disease. Cell 171 (2), 273–285. 10.1016/j.cell.2017.09.021 28985560 PMC5685180

[B29] Tadokoro T. Ikeda M. Ide T. Deguchi H. Ikeda S. Okabe K. (2020). Mitochondria-dependent ferroptosis plays a pivotal role in doxorubicin cardiotoxicity. JCI insight 5 (9), e132747. 10.1172/jci.insight.132747 32376803 PMC7253028

[B30] Urquhart S. Willis S. (2020). Long-acting GLP-1 receptor agonists: findings and implications of cardiovascular outcomes trials. JAAPA official J. Am. Acad. Physician Assistants 33 (8), 19–30. 10.1097/01.JAA.0000669452.63883.45 32740122

[B31] Wang S. Song R. Wang Z. Jing Z. Wang S. Ma J. (2018). S100A8/A9 in inflammation. Front. Immunol. 9, 1298. 10.3389/fimmu.2018.01298 29942307 PMC6004386

[B32] Wang Z. Ma L. Liu M. Fan J. Hu S. Writing Committee of the Report on Cardiovascular Health and Diseases in China (2023). Summary of the 2022 report on cardiovascular Health and diseases in China. Chin. Med. J. 136 (24), 2899–2908. 10.1097/CM9.0000000000002927 38018129 PMC10752444

[B33] Wang Z. Yao M. Jiang L. Wang L. Yang Y. Wang Q. (2022). Dexmedetomidine attenuates myocardial ischemia/reperfusion-induced ferroptosis via AMPK/GSK-3β/Nrf2 axis. Biomed. & Pharmacother. = Biomedecine & Pharmacother. 154, 113572. 10.1016/j.biopha.2022.113572 35988428

[B34] Wu J. Chen S. Liu Y. Liu Z. Wang D. Cheng Y. (2020). Therapeutic perspectives of heat shock proteins and their protein-protein interactions in myocardial infarction. Pharmacol. Res. 160, 105162. 10.1016/j.phrs.2020.105162 32828911

[B35] Wu X. Li Y. Zhang S. Zhou X. (2021a). Ferroptosis as a novel therapeutic target for cardiovascular disease. Theranostics 11 (7), 3052–3059. 10.7150/thno.54113 33537073 PMC7847684

[B36] Wu Y. Liu H. Wang X. (2021b). Cardioprotection of pharmacological postconditioning on myocardial ischemia/reperfusion injury. Life Sci. 264, 118628. 10.1016/j.lfs.2020.118628 33131670

[B37] Xu T. Ding W. Ao X. Chu X. Wan Q. Wang Y. (2019). ARC regulates programmed necrosis and myocardial ischemia/reperfusion injury through the inhibition of mPTP opening. Redox Biol. 20, 414–426. 10.1016/j.redox.2018.10.023 30415165 PMC6230922

[B38] Yang Y. Lin X. (2022). Potential relationship between autophagy and ferroptosis in myocardial ischemia/reperfusion injury. Genes & Dis. 10 (6), 2285–2295. 10.1016/j.gendis.2022.02.012 PMC10404879 37554184

[B39] Yu Y. Yan Y. Niu F. Wang Y. Chen X. Su G. (2021). Ferroptosis: a cell death connecting oxidative stress, inflammation and cardiovascular diseases. Cell death Discov. 7 (1), 193. 10.1038/s41420-021-00579-w 34312370 PMC8313570

[B40] Zhai M. Li B. Duan W. Jing L. Zhang B. Zhang M. (2017). Melatonin ameliorates myocardial ischemia reperfusion injury through SIRT3-dependent regulation of oxidative stress and apoptosis. J. pineal Res., 63(2). 10.1111/jpi.12419 28500761

[B41] Zhang K. Tian X. M. Li W. Hao L. Y. (2023). Ferroptosis in cardiac hypertrophy and heart failure. Biomed. & Pharmacother. = Biomedecine & Pharmacother. 168, 115765. 10.1016/j.biopha.2023.115765 37879210

[B42] Zhang S. Yan F. Luan F. Chai Y. Li N. Wang Y. W. (2024). The pathological mechanisms and potential therapeutic drugs for myocardial ischemia reperfusion injury. Phytomedicine Int. J. phytotherapy Phytopharm. 129, 155649. 10.1016/j.phymed.2024.155649 38653154

[B43] Zhao B. Yu J. Luo Y. Xie M. Qu C. Shi Q. (2023). Deficiency of S100 calcium binding protein A9 attenuates vascular dysfunction in aged mice. Redox Biol. 63, 102721. 10.1016/j.redox.2023.102721 37163872 PMC10189516

[B44] Zhu Q. Luo Y. Wen Y. Wang D. Li J. Fan Z. (2023). Semaglutide inhibits ischemia/reperfusion-induced cardiomyocyte apoptosis through activating PKG/PKCε/ERK1/2 pathway. Biochem. biophysical Res. Commun. 647, 1–8. 10.1016/j.bbrc.2023.01.049 36706596

[B45] Zou Y. Zhang M. Wu Q. Zhao N. Chen M. Yang C. (2022). Activation of transient receptor potential vanilloid 4 is involved in pressure overload-induced cardiac hypertrophy. eLife 11, e74519. 10.7554/eLife.74519 35731090 PMC9224988

