# Supplementary material for: Semaglutide attenuates myocardial ischemia-reperfusion injury by inhibiting ferroptosis of cardiomyocytes via activation of PKC-S100A9 axis
Source: Front Pharmacol. 2025 Mar 20;16:1529652. doi: 10.3389/fphar.2025.1529652 (PMC11965666; doi:10.3389/fphar.2025.1529652)
Supplement: Supplementary file 1 [file Table1.docx]

**Supplementary table 1. Primer list**

| **Primers** |  | **Sequences (5'–3')** |
| --- | --- | --- |
| ZBTB16 -Forward |  | AGTGTAATGGCTGTGGCAAGAA |
| ZBTB16- Reverse |  | TGGTACACTGGTATGGCGAGG |
| MAP3K6-Forward |  | TGCCATTGGTGAAGTATGAAGG |
| MAP3K6-Reverse |  | GCCAGCTTCATGCCAATCA |
| Timp4- Forward |  | TGTCTACACGCCATTTGACTCTTC |
| Timp4- Reverse |  | ACAGTTCTGGTGGTAGTGATGATTC |
| Sult1a1- Forward |  | CTGCCCTTGTCCTTACTCCCTC |
| Sult1a1- Reverse |  | ACCACGACCCATAGGACACTTT |
| Nppb- Forward |  | GCTTCTGCGGCATGGATCTC |
| Nppb- Reverse |  | TCCCAGAGGATAGGAGTGACC |
| cfh- Forward |  | CGCCCTGGATACCGAACACT |
| cfh-Reverse |  | CTGCCAGCCTAAAGGACCCA |
| Mgp-Forward |  | GGCAACCCTGTGCTACGAAT |
| Mgp-Reverse |  | GTAGTCATCGCAGGCCTCTC |
| S100A9-Forward |  | GCATAACCACCATCATCGACAC |
| S100A9-Reverse |  | TGCCATCAGCATCATACACTCC |
| GAPDH-Forward |  | CCTCGTCCCGTAGACAAAATG |
| GAPDH-Reverse |  | TGAGGTCAATGAAGGGGTCGT |
