# Supplementary material for: Semaglutide attenuates myocardial ischemia-reperfusion injury by inhibiting ferroptosis of cardiomyocytes via activation of PKC-S100A9 axis
Source: Front Pharmacol. 2025 Mar 20;16:1529652. doi: 10.3389/fphar.2025.1529652 (PMC11965666; doi:10.3389/fphar.2025.1529652)

**Supplementary Figure1: Rescue tests were conducted to investigate the function of S100A9 in ferroptosis**

(A-B) Western blot detection of S100A9, GPX4, COX2 protein expression in HL-1 cardiomyocytes of the three groups(H/R, H/R+Sem, H/R+Sem+Overexpression S100A9) using β-Actin as internal control. (C) WST-8 method was used to detect total SOD activity. (D-F)Colorimetric method was used to detect reduced glutathione (GSH), cellular ferrous ion ($Fe^{2+}$), and lipid peroxidation marker malondialdehyde (MDA) in the HL-1 cardiomyocytes of the three groups(H/R, H/R+Sem, H/R+Sem+Overexpression S100A9). * $p < 0.05$; * * $p < 0.01$; ** * $p < 0.001$; * ** * $p < 0.0001$ (n=6 per group)

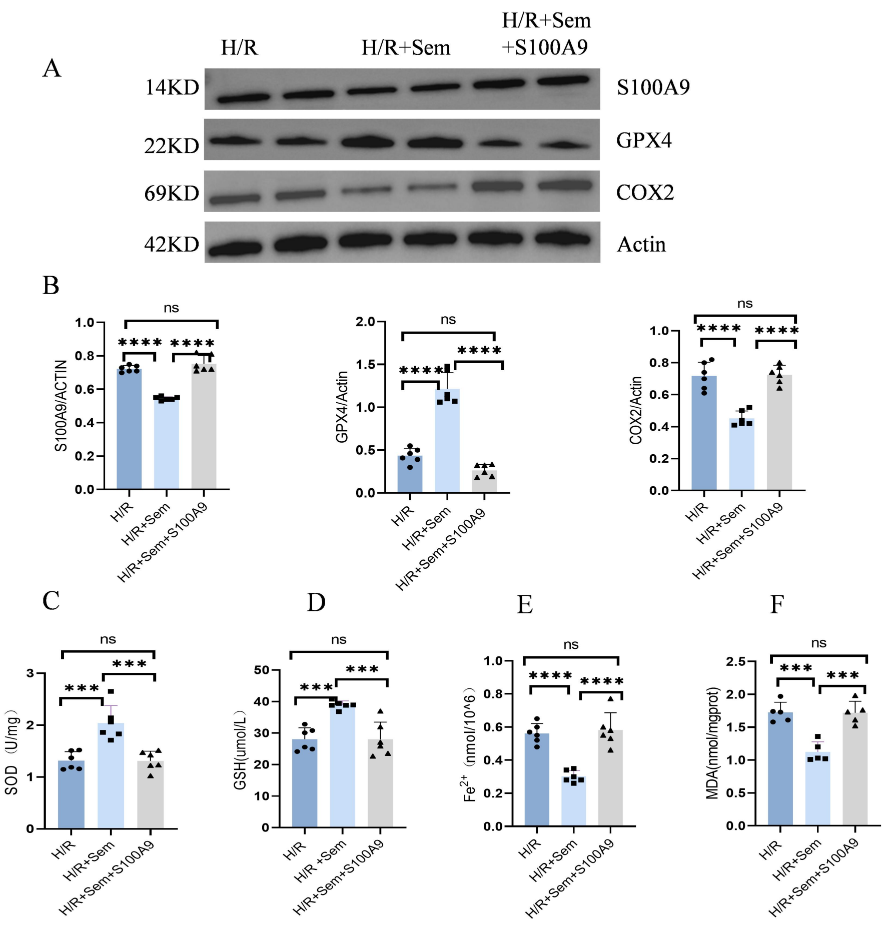

**Supplementary Figure2: Inflammation reaction in MIRI**

(A) Western blot detection of NLRP3 protein expression in HL-1 cardiomyocytes of the three groups (Control, H/R, H/R+Sem) using β-Actin as internal control. (B) IL-6 Elasa kit measured three groups (Control, H/R, H/R+Sem) of IL-6 in Hl-1 cells.

A

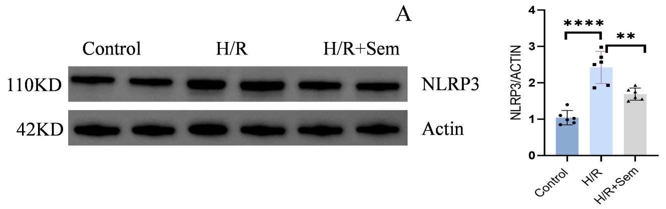


B

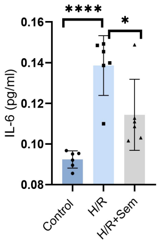

Supplement: Supplementary file 2 [file DataSheet1.pdf]
