# Supplementary figures and images for: Semaglutide attenuates myocardial ischemia-reperfusion injury by inhibiting ferroptosis of cardiomyocytes via activation of PKC-S100A9 axis
Source: Front Pharmacol. 2025 Mar 20;16:1529652. doi: 10.3389/fphar.2025.1529652 (PMC11965666; doi:10.3389/fphar.2025.1529652)

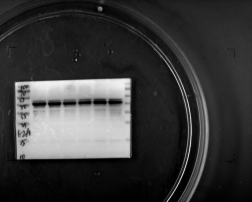

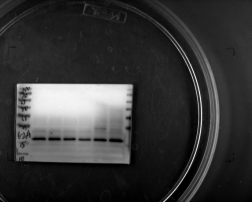

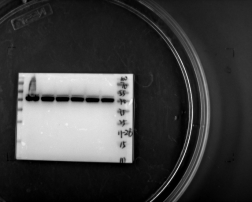

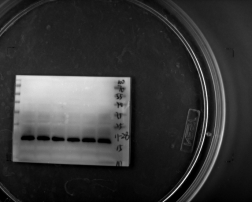


细胞GPX4

动物GPX4


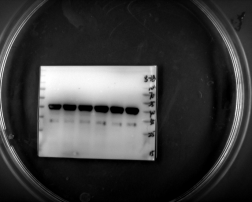

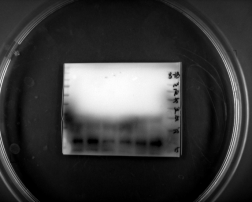

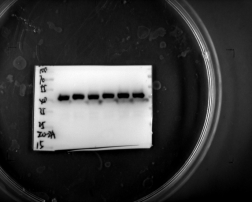

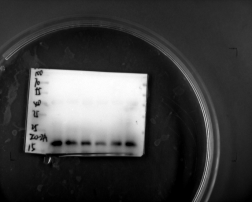


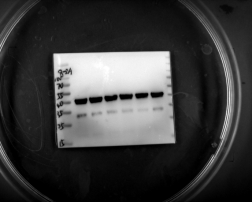

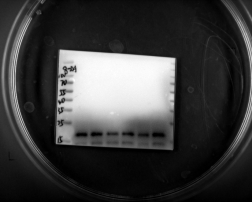

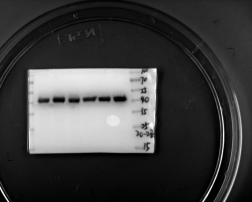

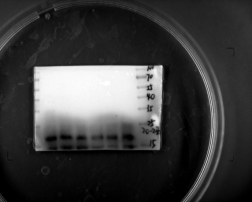


动物COX2

细胞COX2


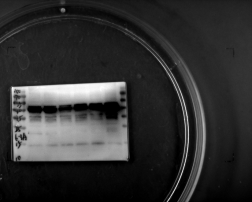

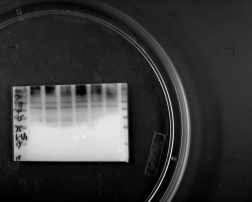

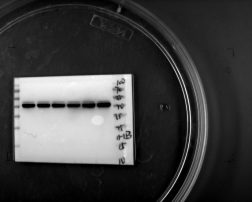

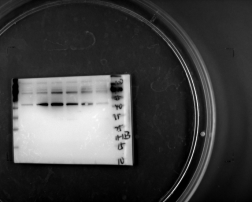


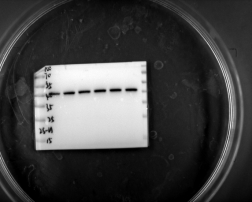

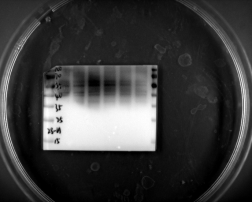

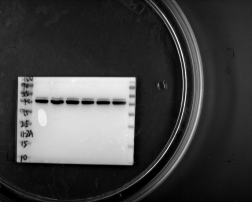

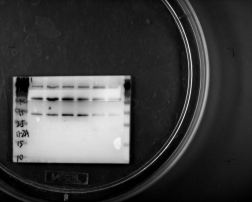


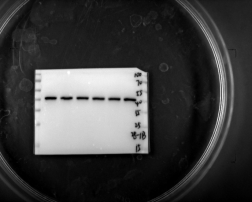

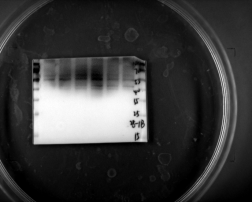

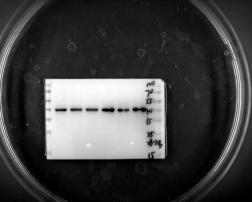

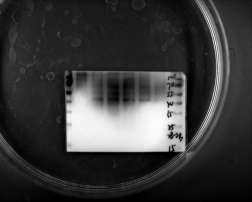


动物S100A9

细胞S100A9


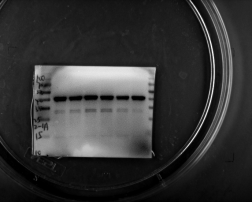

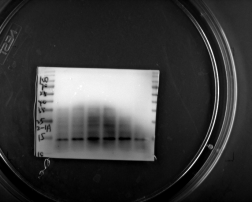

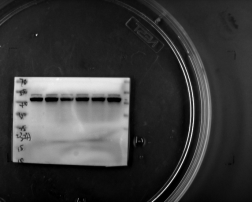

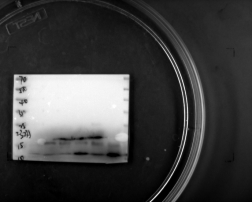


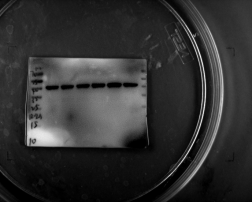

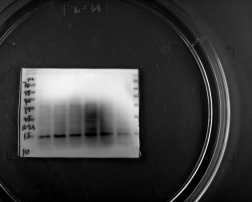

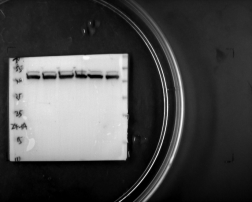

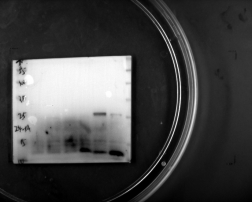


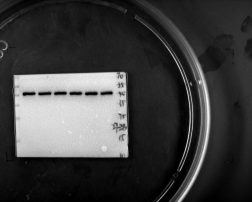

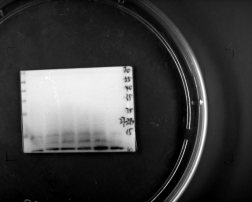

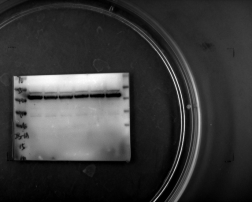

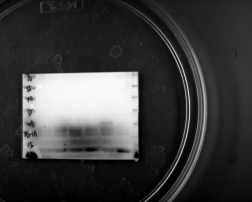


细胞PKC相关

PPKC

PKC


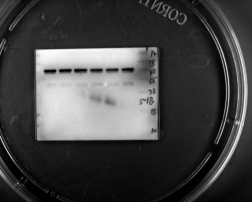

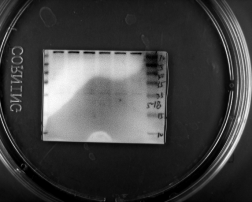

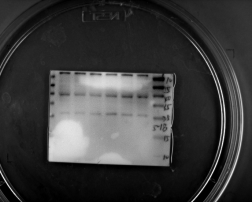


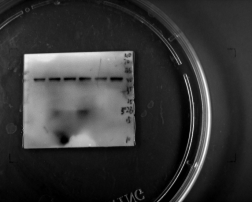

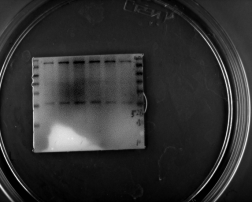

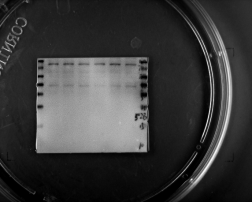


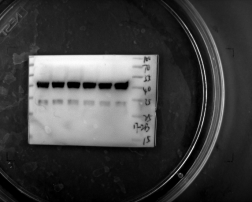

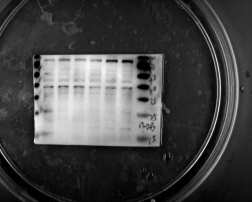

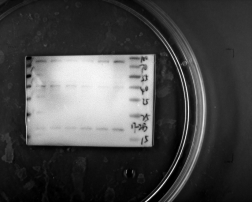


细胞PKC相关

PKC

PPKC


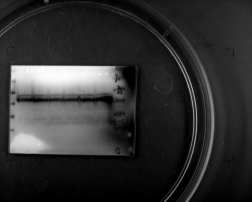

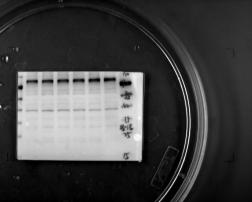

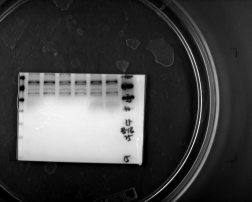


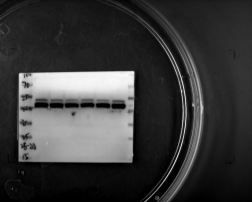

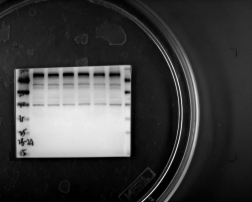

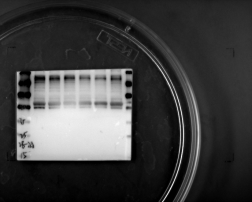


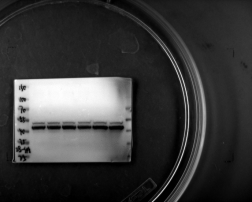

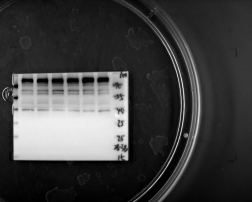

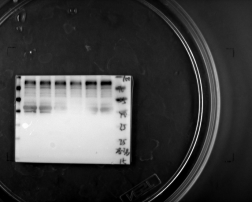

Supplement: Supplementary file 3 [file DataSheet2.docx]
